# Neuroinflammation in early, late and recovery stages in a progressive parkinsonism model in rats

**DOI:** 10.3389/fnins.2022.923957

**Published:** 2022-08-26

**Authors:** Debora M. G. Cunha, Marcela Becegato, Ywlliane S. R. Meurer, Alvaro C. Lima, Narriman Gonçalves, Vinícius S. Bioni, Sheila A. Engi, Paula C. Bianchi, Fabio C. Cruz, Jose R. Santos, Regina H. Silva

**Affiliations:** ^1^Behavioral Neuroscience Laboratory, Department of Pharmacology, Universidade Federal de São Paulo, São Paulo, Brazil; ^2^Behavioral and Evolutionary Neurobiology Laboratory, Department of Bioscience, Universidade Federal do Sergipe, Itabaiana, Brazil

**Keywords:** microglia phenotypes, reserpine, rats, tyrosine hydroxylase, interleukins, catalepsy

## Abstract

Parkinson’s disease (PD) is characterized by motor and non-motor signs, which are accompanied by progressive degeneration of dopaminergic neurons in the substantia nigra. Although the exact causes are unknown, evidence links this neuronal loss with neuroinflammation and oxidative stress. Repeated treatment with a low dose of reserpine—inhibitor of VMAT2—has been proposed as a progressive pharmacological model of PD. The aim of this study was to investigate whether this model replicates the neuroinflammation characteristic of this disease. Six-month-old Wistar rats received repeated subcutaneous injections of reserpine (0.1 mg/kg) or vehicle on alternate days. Animals were euthanized after 5, 10, or 15 injections, or 20 days after the 15th injection. Catalepsy tests (motor assessment) were conducted across treatment. Brains were collected at the end of each treatment period for immunohistochemical and RT-PCR analyzes. Reserpine induced a significant progressive increase in catalepsy duration. We also found decreased immunostaining for tyrosine hydroxylase (TH) in the substantia nigra *pars compacta* (SNpc) and increased GFAP + cells in the SNpc and dorsal striatum after 10 and 15 reserpine injections. Phenotyping microglial M1 and M2 markers showed increased number of CD11b + cells and percentage of CD11b + /iNOS + cells in reserpine-treated animals after 15 injections, which is compatible with tissue damage and production of cytotoxic factors. In addition, increased CD11b + /ArgI + cells were found 20 days after the last reserpine injection, together with an increment in IL-10 gene expression in the dorsal striatum, which is indicative of tissue repair or regeneration. Reserpine also induced increases in striatal interleukin TNF-alpha mRNA levels in early stages. In view of these results, we conclude that reserpine-induced progressive parkinsonism model leads to neuroinflammation in regions involved in the pathophysiology of PD, which is reversed 20 days after the last injection. These findings reveal that withdrawal period, together with the shift of microglial phenotypes from the pro-inflammatory to the anti-inflammatory stage, may be important for the study of the mechanisms involved in reversing this condition, with potential clinical applicability.

## Introduction

Neurodegenerative diseases are characterized by the loss of neuronal populations due to metabolic and toxic changes (Dugger and Dickson, 2017). These alterations lead to crucial events that precede neurodegenerative processes such as oxidative stress and neuroinflammation. Investigating these events is of paramount importance for understanding the pathophysiology of these diseases and identifying promising therapeutic targets.

With the increase in life span, the incidence of aging-related diseases becomes higher. For example, according to an epidemiological study carried out in 2017, Parkinson’s Disease (PD) affects 1–2 people per 1,000 in the general population, reaching 1% in the population over 60 years old (Tysnes and Storstein, 2017). The pathophysiology of PD consists of neurodegeneration of the substantia nigra pars compacta (SNpc), affecting the dopaminergic neurons that send their projections to the dorsal striatum (Tan et al., 2020). Dopamine depletion in the striatum leads to the known motor symptoms of the disease.

Numerous studies with *postmortem*, *in vitro* and non-human animals approaches have shown that neuroinflammation is an important pathway in the PD pathogenesis, and that this process involves both innate and adaptive immunity mechanisms (Thakur and Nehru, 2014; Gelders et al., 2018; Grozdanov and Danzer, 2018). McGeer et al. (1988) were the first to report microgliosis in the brains of PD patients. Subsequently, increases in the gene expression of TNFα and other pro-inflammatory cytokines were found in the brain and cerebrospinal fluid of patients, which led to the “inflammatory hypothesis of neurodegeneration” (Mogi et al., 1994a,b, 1995).

Glial cells play a key role in neuroinflammatory processes. Microglial cells correspond to 10–15% of the central nervous system cells (Mittelbronn et al., 2001) and are resident macrophages from precursor cells of the red marrow. However, unlike peripheral macrophages, these cells cannot be excessively stimulated because they can promote harmful consequences to the neuronal tissue, including death of dopaminergic neurons (Reale et al., 2009).

Among the factors involved in this process, there is the polarization of microglia in M1 or M2 phenotypes in response to a harmful stimulus. M1 is characterized by the production of pro-inflammatory mediators such as interleukins IL1β, IL-6, IL-12, IL-17, IL-18, IL-23, tumor necrosis factor α (TNF-α) and interferon y (INF-y) (Benarroch, 2013; Nakagawa and Chiba, 2015). Nitric oxide induced synthase (iNOS) is one of the membrane markers for this phenotype (Franco and Fernández-Suárez, 2015). M2 phenotype plays a role in immunoregulation, repair and resolution of the inflammatory process, and produces a range of anti-inflammatory mediators such as cytokines IL-4 and IL-10. This phenotype expresses membrane receptors such as protein Arginase I (Arg I); Ym1 (chitinase-like protein), Fizz1 and PPAR (activated peroxisome proliferation receptor) (Subramaniam and Federoff, 2017).

Among the pharmacological animal models used to study PD are those induced by neurotoxins (such as 6-OHDA and MPTP) and by reserpine, an inhibitor of the vesicular transport of monoamines (Leão et al., 2015). In the past, reserpine was used in high doses ranging from 1 to 10 mg/kg, which rapidly induced severe motor impairments (Baskin and Salamone, 1993; Salamone and Baskin, 1996). More recent studies have proposed the repeated administration of 0.1 mg/kg of this drug, which leads to the gradual appearance of motor signs in rats or mice, similar to the condition in humans (Fernandes et al., 2012; Santos et al., 2013; Peres et al., 2016; Campêlo et al., 2017; Leão et al., 2017; Lins et al., 2018; Beserra-Filho et al., 2019; Bispo et al., 2019; Rahman et al., 2020). Furthermore, Santos et al. (2013) demonstrated that animals that received repeated reserpine at low dosages also had cognitive deficits compatible with the non-motor sings of PD. In addition, this protocol also induces reduction in the tyrosine hydroxylase (TH) labeling, increase in lipid peroxidation and increased alpha-synuclein levels in the nigro-striatal dopaminergic pathway (Fernandes et al., 2012; Santos et al., 2013; Leão et al., 2017), which are important factors related to PD pathophysiology. However, neuroinflammation processes possibly related to the alterations induced by this protocol have not been investigated yet.

The aim of this study is to investigate whether the low-dose repeated reserpine model of parkinsonism induces neuroinflammation. Specifically, considering the dopaminergic pathway involved in PD, we investigated immunostaining for astrocytes and microglia markers, the polarization of activated microglia in the M1 (pro-inflammatory) and M2 (anti-inflammatory) microglial phenotypes, and cytokines expression.

## Materials and methods

### Subjects and general procedures

Six-month-old male Wistar rats (*n* = 80) from the Central Bioterium of the Federal University of São Paulo (CEDEME/UNIFESP) were used. All animals were housed under controlled temperature (22–23°C) and lighting (12 h light/12 h dark, with the lights on at 7:30 a.m.) and food and water were provided *ad libidum* throughout the experiment. This project was approved by the local Research Ethics Committee (CEUA N 2790010416) and all procedures were in accordance with the Brazilian law for the use of animals in scientific research (law no. 11794).

Subcutaneous administrations of reserpine or vehicle were performed according to the following experimental groups: animals that received 5 (Res or Veh 5); 10 (Res or Veh 10) or 15 (Res or Veh 15) injections and animals that after the 15th injection remained untreated for 20 days (W/D 20 days) for subsequent euthanasia. Before the beginning of the experimental procedures, the animals were handled for 20 min/day, for 5 consecutive days. Between the experimental sessions, the apparatuses were cleaned with a 5% alcohol solution.

### Drug

Reserpine (Sigma Chemical Co., St. Louis, MO) was dissolved in 100 μL of glacial acetic acid and diluted in distilled water. The control solution contained the same amount of acetic acid and distilled water as the reserpine solution. Vehicle or 0.1 mg/kg reserpine were injected subcutaneously (SC) every other day.

### Experimental design

**FIGURE 1:**
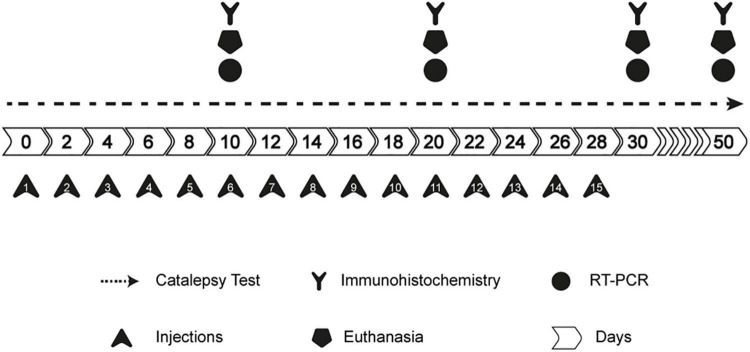
Experimental design.

Animals were randomly distributed in the above-mentioned groups. Injections were administered every other day, and the behavioral evaluations were conducted as shown in Figure 1.

Briefly, the catalepsy test was performed every other day before the next injection. Euthanasia was performed on the 10th, 20th, 30th, and 50th days of the protocol, according to the experimental group. Rats were either transcardially perfused (brains collected for immunohistochemistry) or euthanized by decapitation (striatum dissected and immediately frozen in liquid nitrogen for quantitative PCR) (qPCR).

### Catalepsy test

Each animal was placed with the forepaws resting on a horizontal bar (positioned 9 cm from the surface in which the hind paws were supported). The duration the animal remained in an immobile posture, keeping both forepaws on the bar, was quantified by direct observation up to a maximum of 180 s. This procedure was repeated 3 times on each test day, and the average of the 3 attempts for each animal was considered for analysis.

### Immunohistochemistry

After completion of each treatment duration, the animals were euthanized with an intraperitoneal injection of sodium thiopental (40 mg/kg) and transcardially perfused with 200 mL of phosphate-buffered saline (PBS), pH = 7.4, containing 500 IU of heparin (Roche, Brazil), followed by 300 mL 4.0% paraformaldehyde in 0.1 M phosphate buffer, pH 7.4. Brains were removed and post fixed in the same 4.0% paraformaldehyde solution for 4 h and subsequently transferred to a 30% sucrose solution in 0.1 M PBS, pH 7.4. Brains were serially cut in the coronal plane into 50-μm thick sections with a cryostat (Leica, Germany). The sections were placed sequentially and stored in antifreeze solution. Free-floating sections were washed 4 times (5 min each) in 0.1 M phosphate buffer, pH = 7.4, and incubated for 20 min in a 0.1 M phosphate buffer solution with hydrogen peroxide. After this period, the sections were incubated with a blocker (bovine albumin 2%, Triton X-100 0.3%) for 15 min. Afterward, sections were incubated for 18 h with one of the primary antibodies—anti-TH (Millipore, Billerica, MA, United States, 1: 5,000); anti-GFAP (Millipore, Billerica, MA, United States, 1: 1,000); anti-IBA 1 (Millipore, Billerica, MA, United States, 1: 500). After 18 h, the sections were incubated with a specific secondary antibody for each primary antibody (Vector Labs, 1: 1,000). The sections were again incubated in the avidin-biotin complex (ABC Kit, Vector Labs, United States) for 2 h and then incubated in a 0.05% solution of 3.3′ diaminobenzidine (DAB) (Sigma Company, United States) for 2 min. Subsequently, we added 100 μL of hydrogen peroxide for the deposition of the chromogen. After reaching an adequate level of color, the sections were submitted to a battery of dehydration, and mounted on slides and cover slipped with Entellan (Merck, Darmstadt, Germany).

Photomicrographs of the brain structures of interest—striatum and substantia nigra—were taken using an Olympus Microscope, BX-41, with CCD (Nikon, DXM-1200) camera attached. For each animal, 4 sections of each area were selected (one at the rostral level, two at the medium level, and one at the caudal level). The location of the region was determined based on the Paxinos and Watson rat brain atlas (2009). We delineated SNpc or Dorsal Striatum in ImageJ software and performed manual counting of the marked cells for the whole extension within each section for the analysis of TH immunostaining. Relative optical density (ROI) was used to analyze GFAP and IBA 1 markers. Values were expressed relative to Veh 5 injections group (Santos et al., 2013; Leão et al., 2017).

### Immunofluorescence

After perfusion and post fixation (as described above), brain sections were washed 4 times (5 min) in 0.1 M phosphate buffer, pH = 7.4, and antigenic recovery was performed with 0.01 molar sodium citrate (pH 8.0) in a water bath at 70°C for 20 min. Subsequently, the sections remained at room temperature submerged in the citrate for 20 min. The material was washed 4 times and incubated in phosphate buffer, hydrogen peroxide and 10% methanol, remaining in agitation for 20 min. Sections were again washed and then incubated in 1% Tween 20 (Sigma) together with 1% Bovine Serum Albumin (BSA—Merck) and the primary antibodies, namely anti-CD11b (OX42—CBL1512—1: 400); anti-iNOS (AB5382- 1: 600) and anti-ArginaseI (ArgI—PA5-32267—1: 250) remaining in slow agitation for 42 h. Afterward, the cuts were washed and incubated with respective secondary antibody (Alexa Fluor^®^ 568 and 488—1:500) for 2 h. 1 μL of DAPI (Thermo Fisher Scientific, Waltham, MA, United States) was added during the washing procedure for 5 min and then the sessions were mounted on slides and cover slipped with Vectashield (Vector Laboratories, Newark, CA, United States). Illustrative images were treated for background subtraction using the open bioimage informatics platform Icy (de Chaumont et al., 2012).

### Gene expression of pro-inflammatory cytokines (TNF-α, IL-10, and IL-1β)

The quantification of pro-inflammatory cytokines in the striatum was carried out using RT-PCR. We investigated the expression of TNF-α, IL-10, and IL-1β genes. Total RNA extraction from the samples was carried out using the total RNA purification kit (Cellco Biotec) and subsequently the RNA quantification was performed in the SPECTROstar^®^ Nano (BMG LabTech). The complementary DNA (cDNA) was synthesized from 0.5 μg total RNA using SuperScript™ III First-Strand Synthesis System kit (Invitrogen Life Technologies) according to the manufacturer’s specifications.

Each 15 μL RT-PCR reaction was performed with 2.0 μL cDNA as a template, using the TaqMan^®^ Fast Advanced Master Mix (Applied Biosystems, Thermo Fisher Scientific, Waltham, MA, United States). The RT-PCR assays were performed in duplicates on the Applied Biosystem 7500 Real-Time PCR System using TaqMan probes for TNF-α (Rn00562055_m1); IL-10 (Rn01483988_g1) and IL-1β (Rn00676333_g1). Pde10A (Rn00673152_m1) was used as the endogenous control gene. The samples were subjected to the followed PCR protocol template: 50°C for 2 min, 95°C for 10 min, then 40 cycles with denaturation at 95°C for 15 s and annealing and extension at 60°C for 1 min. Data were collected using 7500 Fast SDS Software version 1.5.1 (Applied Biosystems). Calculations of relative expression from Ct (threshold cycle) data were performed using the formula ΔCt = Ct (target gene) – Ct (Pde10A).

### Statistical analysis

All data were checked for normality using the Kolmogorov-Smirnov test. Subsequently, data were analyzed by the two-way ANOVA. Analyses *a posteriori* were performed using the Sidak’s test or Bonferroni’s test (catalepsy behavior). The results were considered statistically significant at *p* < 0.05. Effect size tests (partial η^2^) were applied to significant results of the immunofluorescence analysis. IBM-SPSS Statistics, version 22.0 and GraphPad Prism 8.0 softwares were used to perform the analyses.

## Results

### Behavioral analysis

#### Bar catalepsy

**FIGURE 2:**
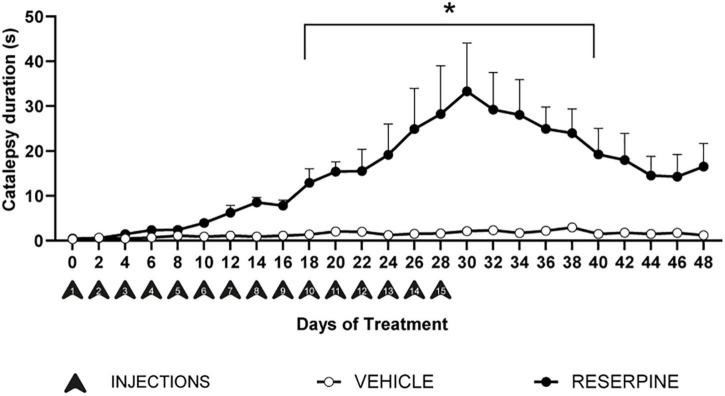
Effects of repeated administration of 0.1 mg/kg reserpine on catalepsy behavior (*n* = 9–10). Data are shown as mean + SEM.; *p < 0.05 comparing Reserpine and Vehicle groups (Two-way ANOVA with repeated measures followed by Bonferroni’s test).

Two-way ANOVA with repeated measures for catalepsy duration considering only the groups that went through the whole protocol revealed effects of treatment [*F* = (1, 425) = 1174.1; *p* = 0.0001]; time [*F* = (24, 425) = 3.591; *p* = 0.0001] and interaction between time and treatment [*F* = (24, 425) = 2.989; *p* = 0.0001]. *Post hoc* analysis with Bonferroni’s test revealed that reserpine-treated group had increase catalepsy duration compared to vehicle from the 10th injection until the 40th day of the protocol (Figure 2). In addition, the mean catalepsy duration in all observations was compared among groups. Two-way ANOVA revealed effects of treatment [*F* = (1, 70) = 30.60; *p* < 0.0001], number of injections [*F* = (3, 70) = 6.596; *p* = 0.0005] and interaction between treatment and number of injections [*F* = (3, 70) = 6.246; *p* < 0.0008]. Sidak’s *post hoc* revealed that Reserpine 15 injections group had increase catalepsy duration compared to all vehicles and all other reserpine-treated groups, as demonstrated in Table 1.

**TABLE 1:** Effects of repeated administration of 0.1 mg/kg reserpine (Res) or vehicle (Veh) on catalepsy test (*n* = 9–10).

Injections	Treatment	Mean catalepsy duration(s)
5 injections	Veh	01.51 ± 0.44
	Res	02.73 ± 0.75
10 injections	Veh	02.20 ± 0.38
	Res	10.72 ± 1.35
15 injections	Veh	02.12 ± 0.52
	Res	33.30 ± 8.28*
W/D 20 days	Veh	01.17 ± 0.28
	Res	16.53 ± 5.17

Data are expressed as mean ± SEM. *p < 0.05 compared to all other groups (two-way ANOVA followed by Sidak’s test).

### Neurochemical analysis

#### Immunohistochemistry for tyrosine hydroxylase

**FIGURE 3:**
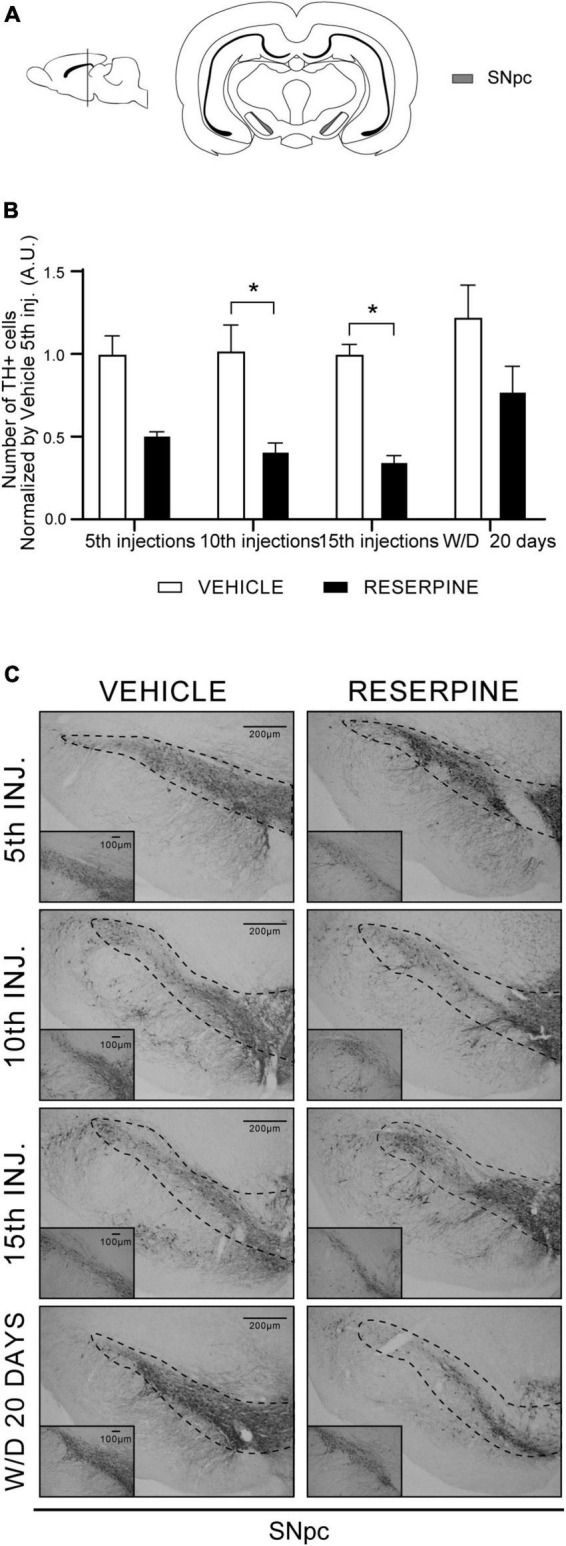
Coronal slice scheme of SNpc **(A)** effects of repeated administration of 0.1 mg/kg reserpine (*n* = 5) on number of TH + cells in the SNpc **(B)** and representative photomicrographs of TH immunostaining in SNpc coronal sections **(C)**. Data are shown as mean + SEM., *p < 0.05 comparing Reserpine and Vehicle groups (ANOVA followed by Sidak’s test). Scale bar in SNpc: 200 μm and magnification 4x in overview images and scale bar of 100 μm further magnified insets (10x).

Two-way ANOVA for TH immunostaining in SNpc revealed effects of treatment [*F* = (1, 28) = 51.21; *p* < 0.0001]. Sidak’s test revealed reduced number of TH + cells in the SNpc region in Res10 (*p* = 0.013) and Res 15 (*p* = 0.003) compared to their respective controls. Res10 injections also differed from the Veh 5 injections (*p* = 0.018) and Veh W/D 20 (*p* < 0.001), as shown in Figure 3. Figure 3C displays representative photomicrographs of TH immunostaining.

#### Immunohistochemistry for GFAP

**FIGURE 4:**
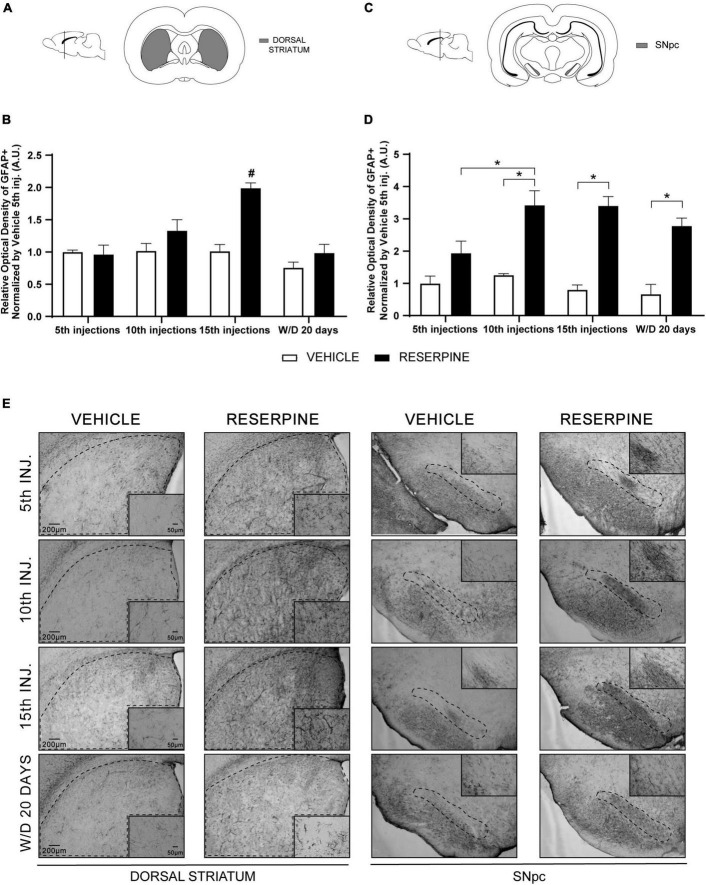
Coronal slice scheme of Dorsal Striatum **(A)** and SNpc **(C)**; effects of repeated administration of 0.1 mg/kg reserpine (*n* = 5) on relative optical density (ROI) of GFAP staining in Dorsal Striatum **(B)** and SNpc **(D)**; and representative photomicrographs of GFAP immunostaining in coronal sections **(E)**. Data are shown as mean + SEM. #*p* < 0.05 comparing Res15 with all Reserpine and Vehicle groups; *p < 0.05 comparing Reserpine and Vehicle groups (ANOVA followed by Sidak’s test). Scale bar 200 μm and magnification 4x in overview images and scale bar of 50 μm further magnified insets (20x).

In the dorsal striatum, two-way ANOVA revealed effects of treatment [*F* = (1, 23) = 18.92; *p* < 0.0002] and number of injections [*F* = (3, 23) = 10.07; *p* < 0.0002], as well as in the interaction between number of injections and treatment [*F* = (3, 23) = 6.622; *p* < 0.0022]. Sidak’s *post hoc* showed that there was a significant increase of ROI in Res15 compared of all reserpine and vehicle groups (Figures 4A,B,E).

Two-way ANOVA analysis for data of the SNpc revealed effects of number of injections [*F* = (3, 23) = 3.459; *p* = 0.0329], treatment [*F* = (1, 23) = 81.30; *p* < 0.0001] and interaction between these factors [*F* = (3, 23) = 3.170; *p* = 0.0435]. Sidak’s *post hoc* showed an increase of ROI in the SNpc after 10 (*p* = 0.0022) and 15 (*p* = 0.0002) injections of reserpine and in the 20th day after withdrawal (*p* = 0.0029) compared to their respective controls. In addition, Res10 group had higher ROI value compared to the Res5 group (*p* = 0.0300) (Figures 4C–E).

#### Immunohistochemistry for IBA 1

**FIGURE 5:**
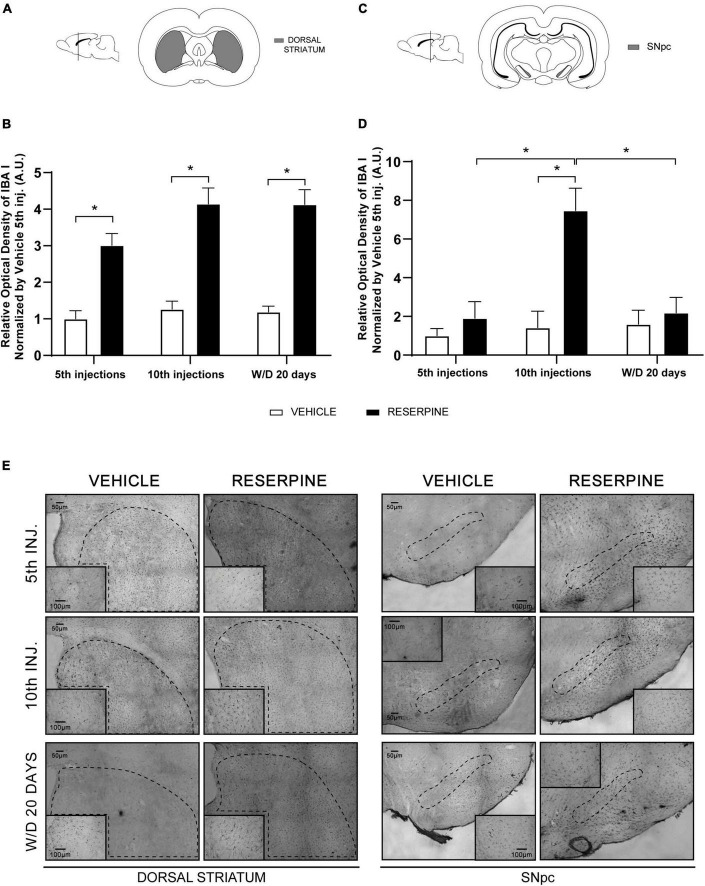
Coronal slice scheme of Dorsal Striatum **(A)** and SNpc **(C)**; effects of repeated administration of 0.1 mg/kg reserpine (*n* = 5) on relative optical density (ROI) for IBA 1 staining in Dorsal Striatum **(B)** and SNpc **(D)**; and representative photomicrographs in coronal sections **(E)**. Data are shown as mean + SEM, *p < 0.05 comparing Reserpine and Vehicle groups (ANOVA followed by Sidak’s test). SNpc: scale bar 200 μm; magnification 4x in overview images and scale bar of 100 μm further magnified insets (10x). Dorsal Striatum: scale bar 300 μm; magnification 2x in overview images and scale bar of 200 μm further magnified insets (4x).

In the dorsal striatum, two-way ANOVA revealed effect of treatment [*F*(1, 21) = 104.1; *p* < 0.0001]. The Sidak’s test revealed increased of ROI in all reserpine groups compared with Veh5. Furthermore, Sidak’s test revealed increased of ROI of Res W/D 20 days injections compared with Veh W/D 20 days injections (*p* = 0.0001), as shown in Figures 5A,B,E.

In the SNpc, two-way ANOVA for IBA I immunostaining revealed effects of number of injections [*F*(2, 21) = 8.012, *p* = 0.0026], treatment [*F*(1, 21) = 14.57, *p* = 0.0010], as well as of the interaction between number of injections and treatment [*F* = (2, 21) = 7.223; *p* < 0.0041]. *A posteriori* analysis using the Sidak’s test showed increased of ROI in the Res10 compared to all reserpine and vehicle groups, as shown in Figures 5C,D.

#### Immunofluorescence for CD11b/iNOS (microglia M1 phenotype)

**FIGURE 6:**
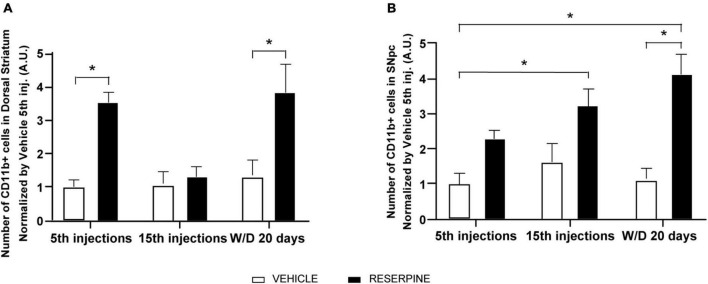
Effects of repeated administration of 0.1 mg/kg reserpine (*n* = 3) on number of CD11b + cells in Dorsal Striatum **(A)** e SNpc **(B)**. Data are shown as mean + SEM, *p < 0.05 comparing Reserpine and Vehicle groups (ANOVA followed by Sidak’s test).

**FIGURE 7:**
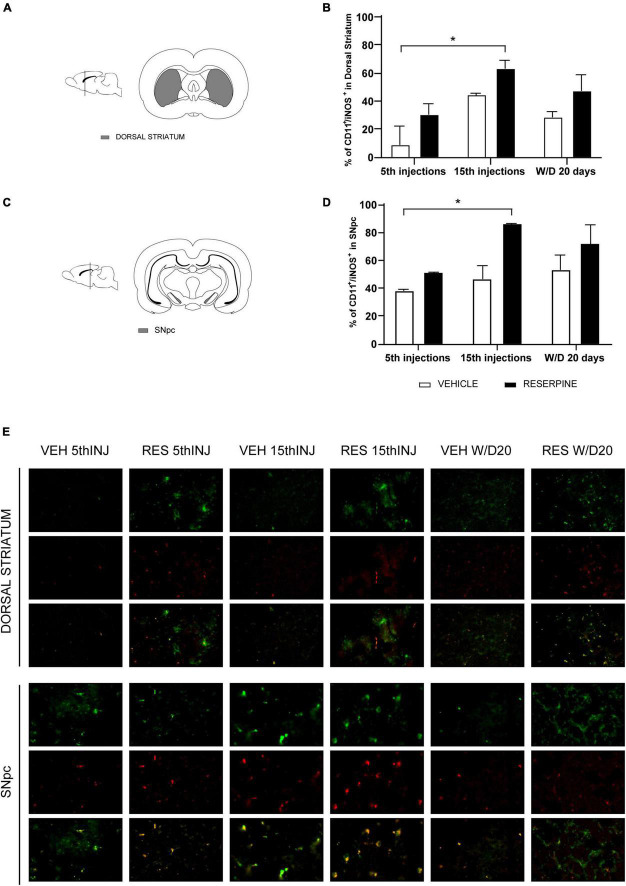
Coronal slice scheme of Dorsal Striatum **(A)** and SNpc **(C)**; effects of repeated administration of 0.1 mg/kg reserpine (*n* = 3) on immunofluorescence with double labeling for CD11b (green) and iNOS (red) expressed by percentage of CD11 + /iNOS + cells in Dorsal Striatum **(B)** and SNpc **(D)**; and representative photomicrographs of immunofluorescence in SNpc coronal sections and Dorsal Striatum **(E)**. Data are shown as mean + SEM, *p < 0.05 comparing Reserpine and Vehicle groups (ANOVA followed by Sidak’s test). Magnification 40x.

In the dorsal striatum two-way ANOVA showed effects of number of injections [*F*(2, 12) = 4.973 *p* = 0.026], treatment [*F*(1, 12) = 21.91, *p* = 0.0005] and interaction between the factors [*F*(2, 12) = 4.086, *p* = 0.044]. In Sidak’s *post hoc* Res5 injections and ResW/D 20 days presented higher number of CD11b + cells when compared to their respective vehicles, as shown in Figure 6A. The Res15 injections group (*p* = 0.0076) showed a higher percentage of CD11b + /iNOS + cells in relation to vehicles. The effect size test indicated a large effect of treatment (η^2^ = 0.54) (Figures 7A,B,E).

Two-way ANOVA for SNpc data showed treatment effect [*F*(1, 12) = 33.01, *p* < 0.0001]. In Sidak’s *post hoc* we found increased number of microglia CD11b + cells in Res15 injections (*p* = 0.041) and Res W/D 20 days (*p* = 0.002) groups compared to Veh 5 (Figure 6B). The Res W/D 20 days injections also had a higher number of CD11b + cells compared to the Veh W/D 20 days (*p* = 0.004) as shown in Figure 6B. Furthermore, Res15 injections group had a higher percentage of CD11b + /iNOS + cells (*p* = 0.033) when compared to the Veh 5 injections in the Sidak’s test. The effect size test indicated a large effect of treatment (η^2^ = 0.46) (Figures 7C–E).

#### Immunofluorescence CD11b/ArgI (microglia M2 phenotype)

**FIGURE 8:**
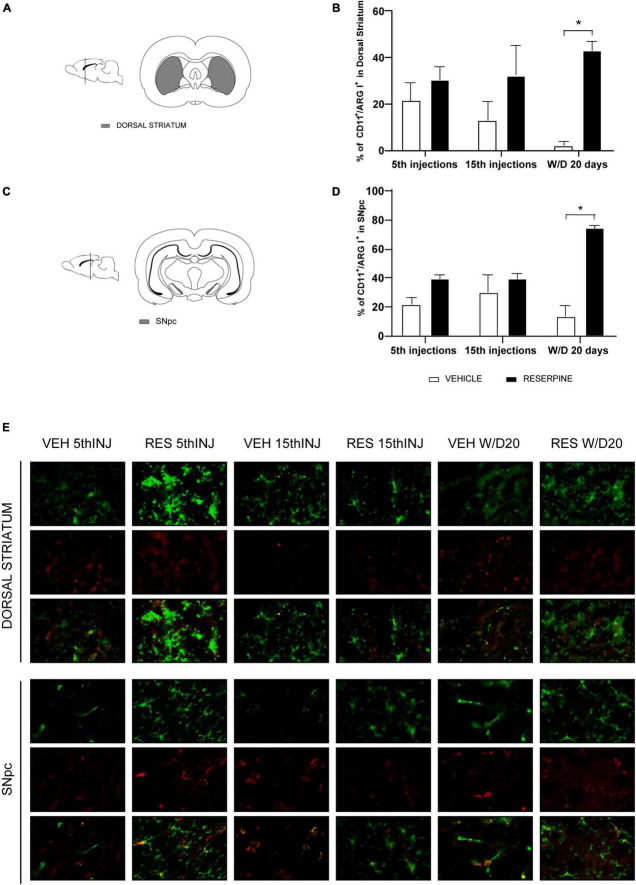
Coronal slice scheme of Dorsal Striatum **(A)** and SNpc **(C)**; effects of repeated administration of 0.1 mg/kg reserpine (*n* = 3) on immunofluorescence with double labeling for CD11b (green) and ArgI (red) expressed by percentage of CD11 + /ArgI + cells in Dorsal Striatum **(B)** and SNpc **(D)**; and representative photomicrographs of immunofluorescence in SNpc coronal sections and Dorsal Striatum **(E)**. Data are shown as mean + SEM, *p < 0.05 comparing Reserpine and Vehicle groups (ANOVA followed by Sidak’s test). Magnification 40x.

In the dorsal striatum, we also obtained an effect of treatment [*F*(1.12) = 13.87; *p* = 0.002] and an increase in the number of CD11b + /ArgI + cells in ResW/D 20 days group when compared to the respective control group (*p* = 0.035). The effect size test indicated a large effect of treatment (η^2^ = 0.71) (Figure 8B). Figure 8D shows the percentage of CD11b + /ArgI + cells in SNpc. Two-way ANOVA revealed effect of treatment [*F*(1.12) = 29.67; *p* < 0.0001] and interaction between factors [*F*(2.12) = 8.906; *p* = 0.004]. *A posteriori* analysis using Sidak’s test revealed an increase in CD11b + /ArgI + cells in ResW/D 20 days group when compared to their respective control (*p* = 0.017). The effect size test indicated a large effect of treatment (η^2^ = 0.54).

#### RT-PCR gene expression of pro-inflammatory cytokines TNF-α, IL-10, and IL-1β

**FIGURE 9:**
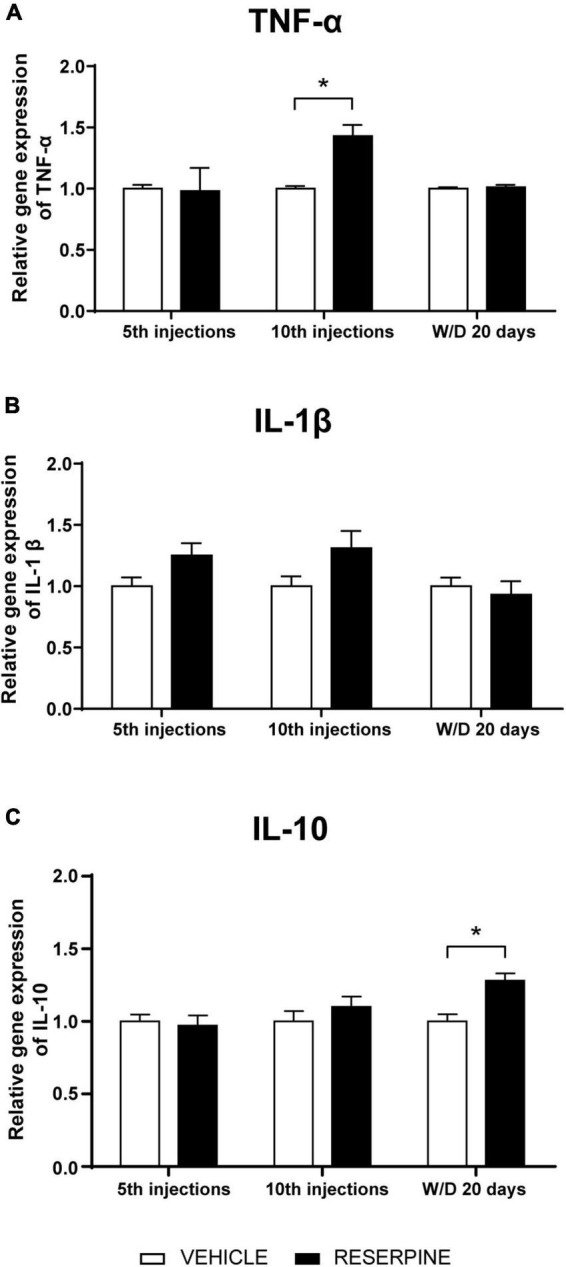
Effects of repeated administration of 0.1 mg/kg reserpine (*n* = 6–8) on gene expression of the cytokines TNF-α **(A)**, IL-1β **(B)**, and IL-10 **(C)** in the dorsal striatum of rats submitted to 5 and 10 injections of reserpine or vehicle and 20 days after the last injection. Data are shown as mean + SEM, *p < 0.05 comparing Reserpine and Vehicle groups (ANOVA followed by Sidak’s test).

Two-way ANOVA for TNF-α gene expression showed effect of treatment [*F* = (1, 48) = 6.713; *p* = 0.012]. Sidak’s *post hoc* revealed an increase in TNF-α gene expression in the Res10 group in relation to Veh10 injections (*p* = 0.042), as shown in Figure 9A. Two-way ANOVA for the IL-1β gene showed a marginal effect of the treatment [*F*(1, 52) = 3.835; *p* = 0.055) (Figure 9B). Two-way ANOVA for IL-10 revealed effect of treatment [*F*(1, 32) = 5.264; *p* = 0.028] and Sidak’s test showed increase gene expression of IL10 in ResW/D 20 days group when compared to the respective control group (*p* = 0.025], as shown in Figure 9C.

## Discussion

The animal model of PD proposed by Fernandes et al. (2012) is induced by repeated administrations of a low dose (0.1 mg/kg) of reserpine. This protocol induces progressive motor changes (catalepsy, decreased spontaneous motor activity and oral dyskinesia), in addition to non-motor signs and neuronal changes compatible with the pathophysiology of PD in both rats (Fernandes et al., 2012; Santos et al., 2013; Sarmento-Silva, 2015; Brandão et al., 2017; Leão et al., 2017; Lins et al., 2017) and mice (Campêlo et al., 2017; Beserra-Filho et al., 2019). Recent adaptations of this protocol have also shown the applicability of reserpine in the study of several aspects of parkinsonism (Pereira et al., 2020; Rahman et al., 2020). In this study, we sought to extend the validation of this protocol, by verifying if neuroinflammation is induced concomitantly to the other alterations related to PD pathophysiology seen in previous studies (decreased dopaminergic markers, increased alpha-synuclein and oxidative stress). Our results reproduced the motor impairment evaluated by the catalepsy test. We demonstrated a progressive increase in the duration of catalepsy, which was statistically significant from the 10th reserpine injection until 12 days after the 15th injection (Figure 2), as well as an increase in the duration of catalepsy in Res 15 injections group compared with all other groups (Table 1). This outcome reinforces that this model mimics the progressiveness of motor decrement and corroborates the results by Santos et al. (2013). However, in that study, only 10 reserpine injections were applied, and 20 days after the 10th injection the duration of catalepsy returned to control levels. Herein, although there was a decline in the duration of catalepsy after the last injection, the difference between the groups remained, showing that the increase in the number of injections precluded the reversion of the motor deficits after treatment interruption. It should be noted that 20 days after the last injection, the increase in catalepsy that is still present (although not statistically significant in the last measures) is hardly due to the acute dopamine depletion caused by each injection of reserpine.

Furthermore, Figure 3 shows a decrease in TH + cells in the SNpc after the 10th and 15th injections, with a partial recovery in withdrawal. This partial recovery corresponds to the reduction in the catalepsy curve in the withdrawal period. These results have also been obtained by other studies with the reserpine model, although there were small differences in protocol and dosage (Santos et al., 2013; de Freitas et al., 2016; Leão et al., 2017). To date, there is no evidence of dopaminergic neuronal death induced by this low-dose reserpine treatment *in vivo*. However, different protocols of reserpine administration induce neuronal effects related to neurodegeneration, such as oxidative stress, α-synuclein and morphology alterations (Abílio et al., 2004; Fernandes et al., 2012; Lee et al., 2015; Reckziegel et al., 2016; Leão et al., 2017). In addition, the neonatal administration of a high dose of reserpine induced neuronal loss and alpha-synuclein brain inclusions (van Onselen and Downing, 2021). Importantly, the irreversibility of neuronal impairment in conventional toxin models has also been questioned. For example, a study showed partial recovery over time after infusion of 6-OHDA (Deumens et al., 2002). In this way, the reversibility of dopaminergic dysfunction after a damaging intervention could be an interesting phenomenon to study potential therapeutic targets.

The above observations support that this protocol of repeated low-dose reserpine administration, despite probably not irreversible, induces long-term changes related to PD. As discussed below, the present study showed that parameters related to neuroinflammation, an important feature of PD pathophysiology, are altered after repeated reserpine treatment.

Increased number of astrocytes in the SNpc and striatum has long been evidenced in PD (Sheng et al., 1993; Hirsch and Hunot, 2009). These cells are over activated by microglia and produce growth factors that enable neuroprotection, and they also participate in the oxidative stress process (Niranjan et al., 2012). In line with these findings, we demonstrated an increase in GFAP + immunoreactive cells in Res10, 15 and ResW/D 20 days in SNpc and in Res15 injections in the dorsal striatum (Figure 4). This result indicates that even 20 days after the end of reserpine administration, inflammation remains in the SNpc, but not in the dorsal striatum, which corroborates both the long-term neuronal changes induced by the treatment and the partial recovery after withdrawal discussed above.

Microglial cells participate in a key pathway of neuroinflammation in neurodegenerative diseases, especially PD (Zhao et al., 2015), and are found in larger quantities in the SNpc compared to other brain areas, even in the healthy brain (Kim et al., 2000). Moreover, this cell type can alternate between neurotoxic and neurotrophic actions, mediating the production of pro or anti-inflammatory cytokines and molecules related to reactive oxygen species (ROS), leading to neuronal death (Trudler et al., 2014). Here, we showed that low-dose repeated reserpine increased immunohistochemical staining of the microglia marker ionized calcium binding adapter molecule 1 (IBA-1), which persisted at least 20 days after treatment withdrawal, in line with other long-term alterations mentioned above (Figure 5).

We also investigated the profile of microglia phenotypes in the different phases of the experimental design. We performed double labeling for activated microglia M1 phenotype using the microglia markers CD11b and iNOS and M2 phenotype using CD11b and ArgI markers, according to previous studies (Zhao et al., 2015; He et al., 2016). We demonstrated an increase in CD11b + microglia in the SNpc of Res15 and ResW/D 20 animals, and in the dorsal striatum in Res5 and ResW/D 20 groups (Figure 6). However, the highest percentage of M1 microglia (CD11b + /iNOS +) was found in the Res15 group for both areas (Figure 7). This reinforces that the period corresponding to 15 reserpine injections shows an established inflammatory process that progressively increased from the beginning of treatment. On the other hand, the results of the CD11b + /ArgI + analysis demonstrated that M2 microglia is significantly higher in SNpc and dorsal striatum only in Res W/D 20 rats (Figure 8). These results are consistent with features of the neuroinflammatory process. Indeed, an insult/injury promotes tissue damage leading to the production of cytotoxic factors such as iNOS, characterizing the M1 (cytotoxic) phenotype (Chhor et al., 2013). The same cell turns to an M2- repair/regenerative phenotype over time (Chhor et al., 2013; Tang and Le, 2016). ArgI catalytic activity produces molecules that repair extracellular matrix and mitochondrial function (Chhor et al., 2013). However, the hypothesis that there is a shift from M1 to M2 microglial phenotype across reserpine treatment and recovery should be considered with caution. Indeed, there are some limitations regarding these analyses. First, due to technical issues, the sample sizes of the immunofluorescence assays are small (although effect size tests indicated large effects for all significant differences). Second, additional M1 and M2 markers would add strong evidence of this claim and should be included in future studies.

Nevertheless, corroborating our observations, an increase in CD11b + /iNOS + microglia was shown in an MPTP model of PD. Rodents submitted to the MPTP model were treated with fasudil, which limits axonal growth, leading to axonal degeneration. As a result, fasudil induces polarization to M2 microglia as well as suppression of inflammatory responses (IL-1β; TNF-α and NF- κB-p65) (Zhao et al., 2015). 6-OHDA led to polarization toward the M1 state in BV2 microglia with increased IL6, IL-1β, TNF-α, and suppressed M2 phenotype (Zhang et al., 2017).

It should be noted that microglial activation is closely associated with aggregated misfolded proteins such as α-synuclein (Rojanathammanee et al., 2011; Tang and Le, 2016). Aggregated α-synuclein released into the extracellular space from dead dopaminergic neurons leads to an increase in reactive oxygen species (ROS) and cytokines (Zhang et al., 2005; Lee et al., 2010). A previous study of our group showed an increase in immunostaining for α-synuclein in the substantia nigra of rats that received 15 injections of 0.1 mg/kg of reserpine (Leão et al., 2017). Herein, we investigated α-synuclein staining after 5 and 10 reserpine injections and in the withdrawal period (see Supplementary material). The results showed an increase in α-synuclein labeling in groups treated with 5 and 10 injections in the SNpc and dorsal striatum when compared to the Veh 5 group. Additionally, in the striatum, the Res W/D 20 days group also had a higher ROI compared to the Veh5 injections. It is possible that the increased TNF-α gene expression in the dorsal striatum after 10 injections found here was related to the α-synuclein peak identified in this same period (Supplementary Figure 1).

We also investigated the gene expression of pro-inflammatory cytokines (TNF-α and IL-1β), which can be produced by both astrocytes and activated microglia in addition to damaged neurons. An increase in TNF-α gene expression was observed after the 10th reserpine injection (Figure 9A). This –result corroborates the fact that this cytokine is usually produced in the early phase of inflammation (Frankola et al., 2011). As described above, with the progression of the neuroinflammatory process, there was subsequent M1 microglial activation (observed in the group that received 15 injections). In the reserpine group, IL-1β did not differ from the control group in the *post hoc* analysis, but there was a marginal effect of the treatment in the two-way ANOVA test (Figure 9B).

Increased proinflammatory cytokines have been reported after treatment with 1 mg/kg reserpine. In that study (Arora and Chopra, 2013), three consecutive days of reserpine administration induced an increase in TNF-α and IL-1β levels in the cerebral cortex and hippocampus. Here, we did not observe changes in the IL-1β gene expression, as mentioned above. However, regarding reserpine effects on neuroinflammation parameters, the present results are unprecedented in the literature due to the analysis of microglia polarization, astrocyte evaluation and the protocol used (low dose, various treatment durations and long-term consequences in the withdrawal period).

We also investigated the gene expression of cytokine IL-10, an anti-inflammatory molecule that acts in periods of tissue repairment. IL-10 gene expression was increased during the withdrawal period (Figure 9C), in accordance with M2 microglia activation in the same period. These data indicate a possible process of CNS restoration following the reserpine-induced inflammatory process. IL-10 has been shown to induce M2 phenotype of microglia or promote the switch from the M1 to M2. Also, this cytokine can suppress iNOS expression by inhibiting NFkB. Interestingly, IL-10 and vitamin D have been tested in pre-clinical trials as possible candidates for a more effective treatment in PD, by reducing the neuroinflammation (Schwenkgrub et al., 2013).

Finally, it is important to mention that due to technical constraints, it was not possible to evaluate all groups in immunofluorescence and in the gene expression assays. In this respect, although the completion of experimental groups in all analyses would be ideal, the lack of some time points does not seem to hinder the general interpretation of the findings. Indeed, as illustrated in Table 2, the peak of the neuronal alteration indicative of neuroinflammation and dopaminergic deficits occur between the 10th and 15th injections, with some of them starting to appear after the 5th injection, even before the emergence of the motor deficit. Overall, these alterations had partial or total recovery 20 days after withdrawal, concomitant to increased anti-inflammatory indicatives. Thus, our results showed that reserpine-induced PD model leads to neuroinflammation, which reinforces the etiological validation of this model and provides tools for the study of this important process in neurodegenerative diseases.

**TABLE 2:** Summary of the results.

Parameter	Figure	5 Res	10 Res	15 Res	W/D 20 days
Catalepsy duration	2	ns	↑	↑	ns
Tyrosine Hydroxylase/SNpc (IH)	3	ns	↑	↑	ns
GFAP/DS (IH)	4	ns	ns	↑	ns
GFAP/SNpc (IH)	4	ns	↑	↑	↑
IBA 1/DS (IH)	5	↑	↑	ni	↑
IBA 1/SNpc (IH)	5	ns	↑	ni	ns
CD11b/DS (IH)	6	↑	ni	ns	↑
CD11b/SNpc (IH)	6	↑	ni	↑	↑
CD11b + iNOS/DS (IF)	7	ns	ni	↑	ns
CD11b + iNOS/SNpc (IF)	7	ns	ni	↑	ns
CD11b + Arg I/DS (IF)	8	ns	ni	ns	↑
CD11b + Arg I/SNpc (IF)	8	ns	ni	ns	↑
TNF-α/DS (RTPCR)	9	ns	↑	ni	ns
IL-1β/DS (RTPCR)	9	ns	ns	ni	ns
IL-10/DS (RTPCR)	9	ns	ns	ni	↑
α-synuclein/DS (IH)	Suppl.	↑	↑	ni	↑
α-synuclein/SNpc (IH)	Suppl.	↑	↑	ni	ns

Outcomes after 5, 10, or 15 injections of reserpine (Res) or after 20 days of treatment withdrawal (W/D 20 days) compared to vehicle-treated groups.↑, increase; ↓, decrease; ns, no significant effect; ni, not included in the analysis; DS, dorsal striatum; SNpc, substantia nigra pars compacta; IH, immunohistochemistry assay; IF, immunofluorescence assay.

## Data availability statement

The raw data supporting the conclusions of this article will be made available by the authors, without undue reservation.

## Ethics statement

The animal study was reviewed and approved by the Comissão de ética no uso de animais—Universidade Federal de São Paulo.

## Author contributions

DC and RS designed the study. DC coordinated and performed the experiments, conducted statistical analysis, and wrote the manuscript. MB, YM, AL, NG, VB, and JS participated in the data collection. SE, PB, and FC coordinated and participated in RT-PCR procedures. VB and JS participated in Figures design. JS and YM contributed to technical standardizations and theoretical discussions. RS coordinated the study and revised the final version. All authors contributed to the article and approved the submitted version.
