# Supplementary material for: Neuroinflammation in early, late and recovery stages in a progressive parkinsonism model in rats
Source: Front Neurosci. 2022 Aug 26;16:923957. doi: 10.3389/fnins.2022.923957 (PMC9459164; doi:10.3389/fnins.2022.923957)
Supplement: Supplementary file 1 [file Data_Sheet_1.PDF]

Supplementary Material

1 Supplementary Data

Additional immunohistochemistry analysis was performed for α-synuclein marker (Santa Cruz 1:500) in dorsal striatum and substantia nigra. Photomicrographs were taken using Olympus Microscope, BX-41, with CCD (Nikon, DXM-1200) camera attached. For each animal, 8 sections of each area were selected. Relative optical density (ROI) was obtained by ImageJ software. Subsequently, the results were normalized by the Veh 5 group and statistically analyzed.

Two-way ANOVA for α-synuclein immunostaining in SNpc revealed effects of treatment [F= (1,21) = 36.76; p <0.0001]. Sidak's test revealed an increase of ROI in the SNpc in Res 5 [p= 0.0139] and Res 10 [p= 0.0009] compared with Veh5 group, as shown in Supplementary FIG 1 C and D. FIG3 E displays representative photomicrographs of α-synuclein immunostaining. In the dorsal striatum, two-way ANOVA revealed effects of treatment [F= (1, 22) = 40.14; p <0.0001]. Sidak's post-hoc showed that there was a significant increase in ROI in Res 5 [p=0.025], 10 [p=0.002] and Res W/D [p=0.008] groups compared with Veh 5 (Supplementary FIG 1 A, B and E).

2 Supplementary Figure Legend

Supplementary Figure 1. Coronal slice scheme of Dorsal Striatum (A) and SNpc (C); effects of repeated administration of 0.1 mg/kg reserpine (n=4-5) on relative optical density (ROI) in Dorsal Striatum (B) and SNpc (D); and representative photomicrographs of α-synuclein immunostaining in coronal sections (E). Data are shown as mean + SEM, * p <0.05 comparing Reserpine and Vehicle groups (ANOVA followed by Sidak's test). Scale bar 200 μm and magnification 4x.
