# Supplementary figures and images for: Neuroinflammation in early, late and recovery stages in a progressive parkinsonism model in rats
Source: Front Neurosci. 2022 Aug 26;16:923957. doi: 10.3389/fnins.2022.923957 (PMC9459164; doi:10.3389/fnins.2022.923957)

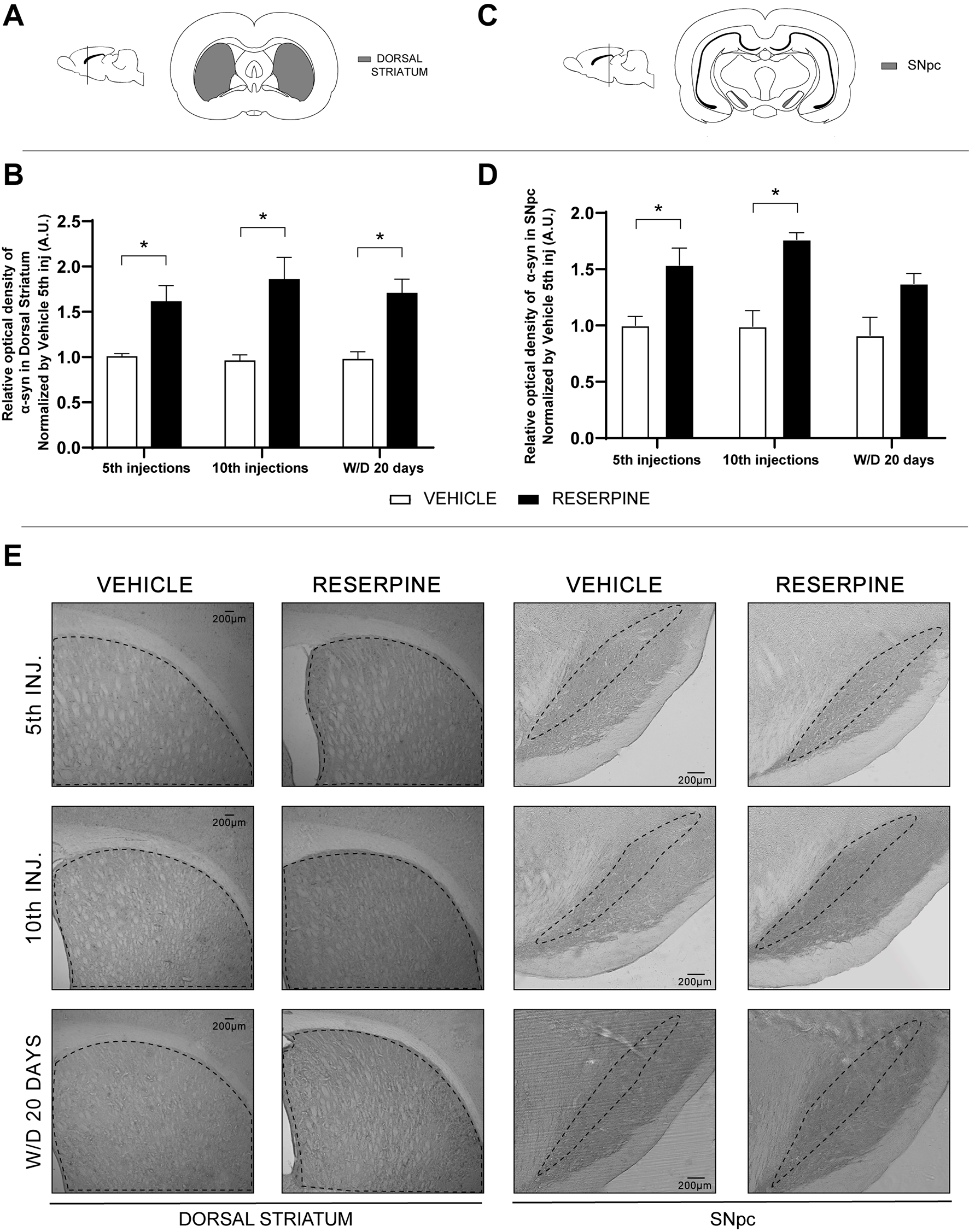

Supplement: Supplementary file 2 [file Image_1.TIF]
